# ECMO-assisted resection of left main bronchial malignant tumor and left pneumonectomy with comprehensive nursing support: a case report

**DOI:** 10.1186/s13019-020-01348-z

**Published:** 2020-10-06

**Authors:** Hui Yang, Ruiyun Chen, Jingru Chen, Fan Yan, Hongmei Zhang, Li Wei, Xiangbo Jia, Yuming Wang

**Affiliations:** 1 Department of Thoracic Surgery, Henan Provincial People’s Hospital, Zhengzhou University People’s Hospital, Henan University People’s Hospital, Zhengzhou, Henan, 450003 China; 2 Nursing Department, Henan Provincial People’s Hospital, Zhengzhou University People’s Hospital, Henan University People’s Hospital, Zhengzhou, Henan, 450003 China; 3 Department of Administration, Henan Provincial People’s Hospital, Zhengzhou University People’s Hospital, Henan University People’s Hospital, Zhengzhou, Henan, 450003 China

**Keywords:** Primary bronchial malignancy, Extracorporeal membrane oxygenation, Left pneumonectomy, Nursing care, Case report

## Abstract

**Background:**

Patients with Extracorporeal Membrane Oxygenation (ECMO) undergoing primary bronchial malignancy resection and left pneumonectomy via bilateral thoracic approach are rare for there exist few reports available to date. And the nursing experience about this disease is rare reported.

**Case presentation:**

This study reported a 50-year-old man with adenoid cystic carcinoma in left main bronchus by computed tomography (CT), fiberoptic bronchoscopy, and puncture biopsy. The case is the first report about operation method and the comprehensive nursing care, including conventional nursing, airway management, fluid management, nutritional support, and psychosocial support for patients undergoing primary bronchial malignancy resection and left pneumonectomy. After multidisciplinary treatment and comprehensive care, the patient was cured and discharged on the 17th day after surgery.

**Conclusion:**

This study reported a rare case with bronchial malignancy resection and left pneumonectomy and discussed its nursing care. A skilled management of ECMO, intraoperative position transformation, and prevention, as well as control of pulmonary complications are fundamental in caring patients with bronchial tumors. Monitoring of pulmonary function and blood pressure, adequate nutrition, and psychological support could be contributing factors for successful treatment during the postoperative stage.

## Introduction

Primary bronchial malignancy refers to a tumor in the annular soft tissue. Malignant tumors between bone and carina that are common to the junction of the cartilage ring with the membranous part [1]. It is relatively rare, occurring in only one in a million of all malignancies [2]. Patients with primary bronchial malignancy may suffer from cough, dyspnea, chest pain, hemoptysis, etc. The diagnosis of bronchial malignancy is primarily based on fiberoptic bronchoscopy and computed tomography (CT) of the chest. The pathological types included squamous cell carcinoma (SCC) and adenoid cystic carcinoma (ACC) [3]. Surgery is currently the most effective treatment of primary bronchial malignancy [4]. However, the existing literature fails to provide standards of indications and surgical treatment for primary bronchial malignancy, and there is still a lack of a standardized approach to treatment [5]. This study sheds new light on surgery as well as comprehensive nursing care for patients with bronchial malignancy. It is expected that this report will provide a useful reference for future bronchial malignancy treatment.

## Case presentation

A 50-year-old male patient was admitted to our hospital for thoracic surgery on March 24, 2020, because of chest tightness and shortness of breath for more than 6 months. The symptoms of chest tightness were not obvious when the patient was calm, but worsened when he was active 6 months ago, the results of the CT scan showed no abnormality at the local hospital. The symptoms of the patient was not relieved after taking antimicrobial drugs (the specific drug name and dosage were unknown).

**Fig. 1:**
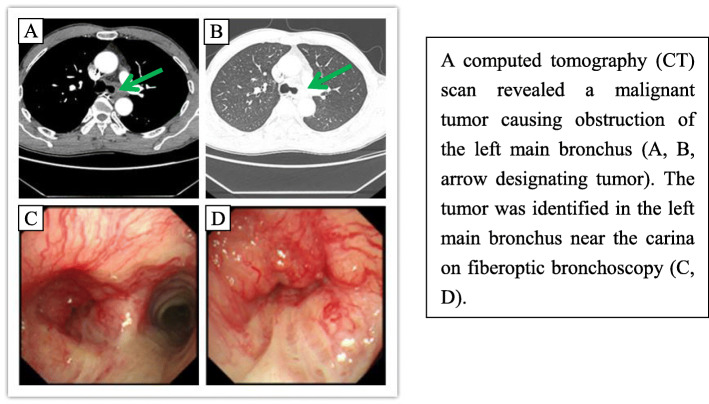
CT and fiberoptic bronchoscopy before operation

The patient was admitted in our department for further treatment. There existed no obvious abnormality on physical examination, but fiberoptic bronchoscopy and puncture biopsy revealed an adenoid cystic carcinoma in the left main bronchus on March 25, 2020 CT (Fig. 1). Because the tumor invaded the carina and inferior segment of the trachea, and the large tumor body located behind the major cardiac vessels, the left lung function of the patient was almost lost. But the coronary angiography showed no obvious abnormality. The electrolytes, serum chemistry, liver and kidney function tests, clotting studies, electrocardiogram, immunohistochemistry, and other relevant tests were performed before the operation. The patient reported no surgery history but a history of smoking and drinking for almost 25 years.

Low-dose oxygen therapy was given to relieve the symptoms of dyspnea, and atomization inhalation, antiasthmatic, expectorant, and antitussive drugs were administered intravenously to relieve cough and sputum symptoms. Considering the risk of traditional left thoracic approach cause by aorta block, the oncology, radiotherapy, intensive medicine, anesthesia, and pathology department were combined for multidisciplinary consultation. A sequential right-left bilateral thoracic approach was chosen for the surgery after discussion. On April 2, 2020, the patient underwent a thoracoscopic-assisted and ECMO-supported right-left sequential bilateral thoracic approach to the lower tracheal carina and left pneumonectomy for lung cancer radical surgery, pleural adhesion, and cauterization, as well as fenestration of pericardium under general anesthesia. During the operation, the VV-ECMO (veno-venous extracorporeal membrane oxygenation) mode of the femoral vein and internal jugular vein was used. Firstly, the patient was under a right lateral position, then the carina and the lower segment of the trachea were dissociated, and 3 cm above the carina, the lower segment of the trachea was separated. After the right main bronchus was fully exposed, the lower position of it was severed from the carina. The 4-0PDS (Polydioxanone) line was used to anastomose the trachea with the right main trachea to ensure the continuity of trachea. After anastomosis, no air leakage was found in the water test, and lung swelling was good, then one thoracic tube was retained. Secondly, the patient was shifted to the left lateral position, and separated the left superior pulmonary vein and the left pulmonary artery trunk in sequence, while the pulmonary artery trunk was blocked for 10 min. The proximal end of the pulmonary artery was cut off when respiratory circulation was stable. After ligated the distal then cutt off the blood vessel, the left pulmonary vein and the left main bronchus, and finally taken out of the left lung specimen. With one thoracic closed drainage tube in a clamped state.

**Fig. 2:**
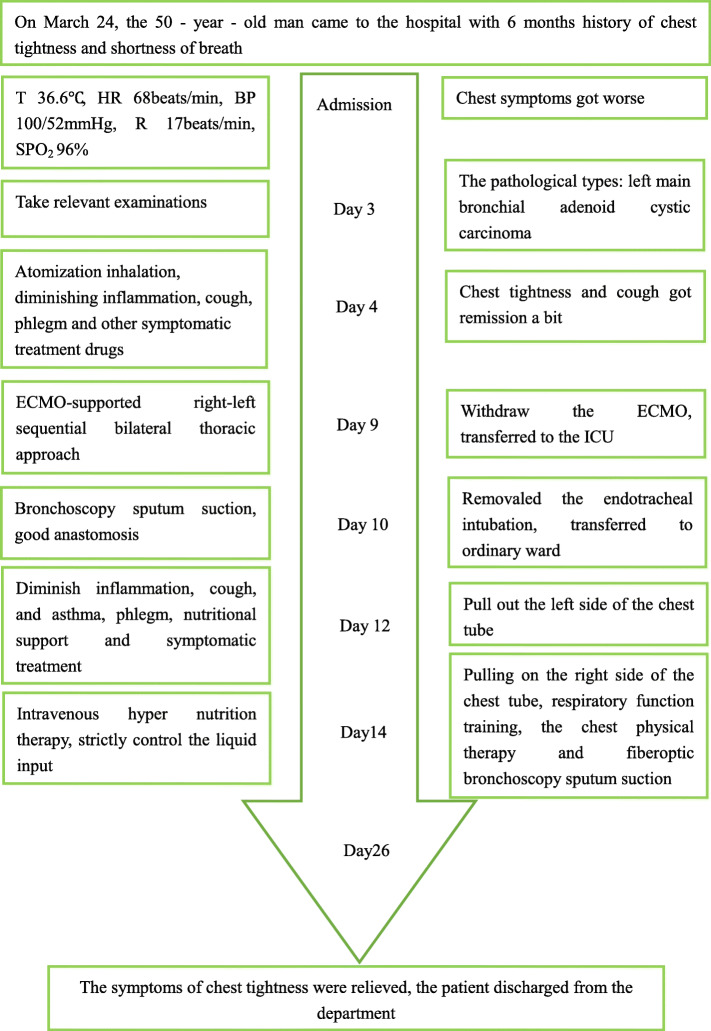
Timeline of the case report. *Notes: T (Body Temperature); HR (Heart Rate); BP (Blood Pressure); R (Breathe); SpO*_*2*_ *(Oxygen Saturation)*

The ECMO was evacuated with the left and right thoracic drainage tubes closed, when the respiratory circulation was stable, and transferred to ICU (Intensive Care Unit) after central venous catheterization and endotracheal intubation. An invasive ventilator was used for ventilation, and the tracheal intubation was removed on April 3, 2020, when the patient’s condition was stable. Then the patient was transferred from ICU to thoracic surgery, and received continuous ECG (electrocardiogram) monitoring and nasal catheter oxygen therapy for 2 L/min. The symptomatic support treatment, such as anti-infection, antitussive, expectorant, analgesic, parenteral nutrition were given, and the liquid drip rate was closely monitored. Considering his age and bedridden time, anticoagulant therapy was given to him. The patient discharged safely on the 17th day after surgery (Fig. 2).

## Discussion

### Conventional nursing

Conventional nursing care played an important role in nursing practices. Pulmonary infection was demonstrated to be the highest mortality complication because of early or delayed treatment for ARDS (Acute Respiratory Distress Syndrome) after pneumonectomy. It was essential to clear up airway secretions timely to prevent lung infection caused by weak cough and sputum. Effective respiratory function training played a significant role in improving patients’ pulmonary function and preventing postoperative pulmonary infection [6]. There needed some precautions for removing secretions after the operation: (1) The nurse instructed the patient to exercise respiratory function in bed every 2 h, to ensure him cough and expectorate effectively. (2) On the first day, the right main bronchus and subordinate lobar bronchus were cleaned with a fiberoptic bronchoscope and normal saline irrigation and aspiration were performed to remove the bloody viscous secretion. Then visible fiberoptic bronchoscopy was performed every 2 days to aspirate sputum, which effectively improved the symptoms of pulmonary infection. (3) The patient’s weak constitution and excision of the carina, leaded to the poor cough and sputum after surgery. In addition, the adequate drainage of the respiratory secretions were not able to be guaranteed, for the limited activity of respiratory cilia caused by the negative balance. The nurse was encouraged to pat the back and guide the patient to cough and expectorate every 2 h without affecting the patient’s rest. The nurse assessed patient’s cough-induced pain, monitored the nature, volume, and color of sputum. Furthermore, evaluating the pulmonary function daily was essential to prevent complications, such as bronchopleural fistula and pneumothorax.

### Nursing essentials and challenges

#### Nursing of ECMO and position transformation

ECMO is the best treatment to replace the cardiopulmonary function and an excellent choice to treat intrabronchial primary bronchial tumors near carina [7]. Due to the special location of the patient’s tracheal tumor, the left lung function was lost. Firstly, doctors resected carina and reconstructed the bronchial of the lower end of the bronchus through the right and left sequential bilateral thoracic cavities, and then carried out left pneumonectomy through the left thoracic cavity. As this operation program was the first case in China, both EMCO nursing and posture conversion were important. The insertion of the ECMO pipeline might promote and consume coagulation and anticoagulant components because it would cause many inflammatory reactions [8]. The medical team decided to use low-dose heparin for anticoagulation at 10 ~ 50 U/kg per hour to maintain Activated Clotting Time (ACT) of 160 s, for the 200 ml intraoperative hemorrhage. At the same time, 4 U of red blood cells and 600 ml of fresh frozen plasma were infused.

ECMO was the essential requirement for protecting the patient’s life, while the position of the patient’s body should be adjusted during the surgery. Considering the potential ineffectively operation, unstable hemodynamics, and pipeline prolapse risks during the position of turning and moving, the teamwork was necessary [9]. A team was formed which included ICU doctors, thoracic surgeons, anesthesiologists, and nurses in the operation theatre, then the team worked out the standard of the position, turning and moving in advance to predict the potential problems while formulating the emergency response measures. After fully assessed the risks and identified the demand of the patient’s position, the anesthesiologist stood next to the patient’s cephalic side to ensure the ventilation of the artificial airway. Meanwhile, an ICU doctor stood at the end of the bed to fix the patient’s lower limbs and observed various parameters of ECMO machines to ensure the blood flow. A thoracic surgeon was in charge of the the ECMO tube. Two surgeons were responsible for moving the waist and buttocks, standing on both sides of the patient. The nurse in operating room supervised the unobstructed infusion pipeline and observed various parameters of the monitor on the infusion side. After the anesthesiologist issued the order, the team successfully changed the patient’s position to the right side without any adverse reactions.

#### Airway management

**Fig. 3:**
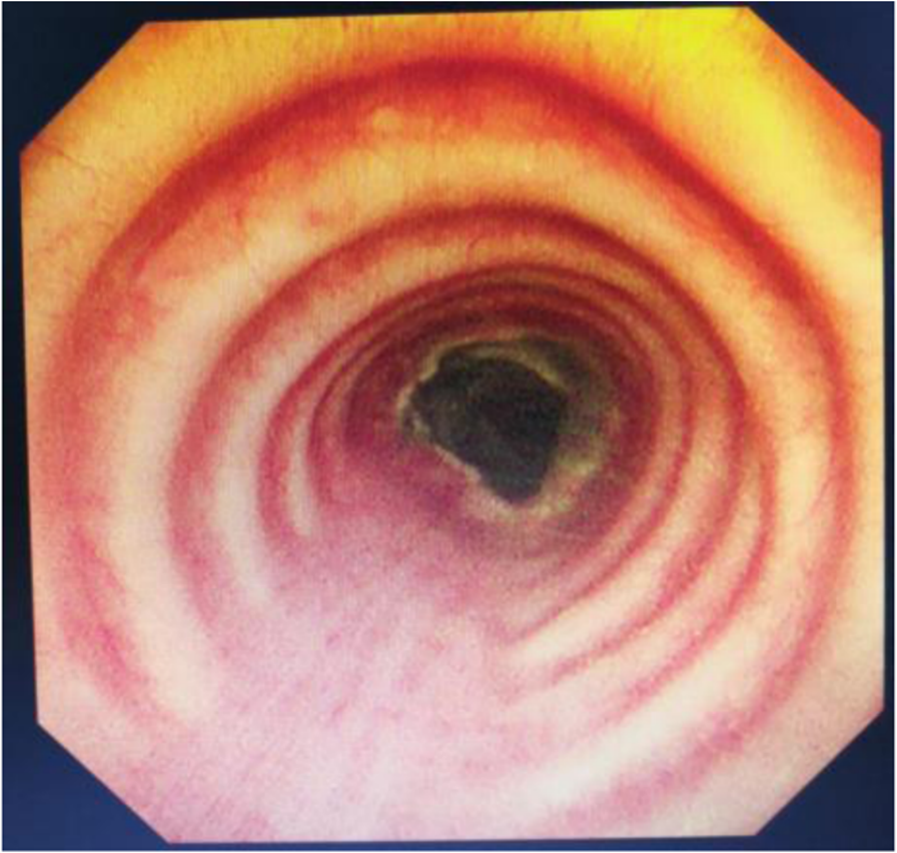
Postoperative bronchial anastomosis

Lung function recovery was the key to the success of this operation, so nursing care should focus on airway management. Expert consensus on perioperative airway management in thoracic surgery (2012 edition) pointed out that airway inflammation was a key link of postoperative pulmonary complications, and was very important to formulate all-round prevention of pulmonary complications during the perioperative process [10]. However, respiratory hypofunction was a challenge after the left pneumonectomy: (1) The “conveyor belt” of the tracheal mucosa-mucus system of the patient was inhibited, which affected the discharge of respiratory secretions; (2) It was easy for blood to flow into bronchus during tracheal anastomosis; (3) Bronchial anastomotic edema after the operation blocked secretion discharge. (4) The disappearance of normal cough reflex after removing the carina, and the sputum tended to accumulate in the airway, and sputum drainage became difficult [11, 12]. Therefore, timely removal of respiratory secretions, resolution of early excretion of sputum, and maintenance of airway patency were critical points of postoperative care. After the operation, the patient was transferred to ICU with a ventilator. The ventilator mode MAQUET was changed to PS/CPAP (personality support/Continuous Positive Airway Pressure), FiO_2_ (Fraction of inspiration O_2_) 35%, PEEP (positive end-expiratory pressure) 5cm H_2_O. Fiberoptic bronchoscopy was performed at the bedside on the first day after the operation. A blood scab was found at the distal end of the tracheal intubation and the tracheal mucosa. After suction, the bronchial was unobstructed, and the tracheal anastomosis was fine (Fig. 3). Besides, a small amount of bloody viscous secretion and brown secretion were extracted from the right bronchus.

The patient was transferred to the general ward of thoracic surgery after removing the tracheal cannula. During the COVID-19 (Corona Virus Disease 2019) pandemic, it was necessary to offer single room isolation for the patient to prevent respiratory complications. The patient was guided to implement respiratory function training, while under oxygen therapy via a nasal catheter. According to the ancient medical theory of traditional Chinese medicine, it was useful to pat the back to promote cough and expectoration at 07: 00, 11: 00, and 19: 00, because these time points were most likely to produce sputum [13]. Atomization inhalation, cough relieving, and phlegm eliminating drugs were applied according to the doctor’s advice. On the third day after surgery, the patient showed an invalid and weak cough, and complained of chest tightness, shortness of breath (SpO_2_ was 88%), caused by the sputum retention and sticky sputum in the small trachea. The respiratory therapist immediately aspirated sputum through bedside fiberoptic bronchoscopy to remove respiratory secretions and relieve the symptoms of the patient.

#### Fluid management

Fluid management was a challenge for patients after operation, in order to maintain stable circulation, ensure organ perfusion, avoid or reduce pulmonary edema, and reduce the burden on heart and lungs. Appropriate restriction of liquid intake was beneficial to improve postoperative oxygenation and promote the recovery of lung function [14]. Swartz et al. [15] reported that the mortality of cardiopulmonary complications was only 2.6% when the fluid volume was less than 1000 ml in the first 12 h after the operation. When the fluid volume exceeded 1000 ml, the mortality rose sharply to 17.3%. The patient received 1095 ml of intravenous infusion within 24 h on the first postoperative day, with a total output of 1270 ml (820 ml of urine and 450 ml of right thoracic drainage) and a total balance of − 175 ml. The excessive intravenous infusion might result in pulmonary edema, whereas insufficient volume might lead to tissue hypoperfusion. We strictly controlled and stabilized the infusion, and the pump speed was set to 100 ml/h. The patient suffered from arrhythmia due to low blood volume with a heart rate of 148 beats/min, then amiodarone injection of 0.3 g and 5% glucose injection of 44 ml were pumped with a micropump of 7 ml/h rate. The patient’s heart rate recovered to 106 beats/min 30 min later. Amiodarone was given last for 7 days after surgery. The urine volume was monitored and maintained at 0.5 ~ 1.0 ml/kg by a comprehensive assessment of the patient’s previous blood pressure level and effective circulation rate. Moreover, nurses evaluated and managed the patient’s fluid balance and effective circulation volume per shift to ensure the fluid intake and output balance or relative negative balance to minimize pulmonary edema [16].

#### Nutrition support

**Fig. 4:**
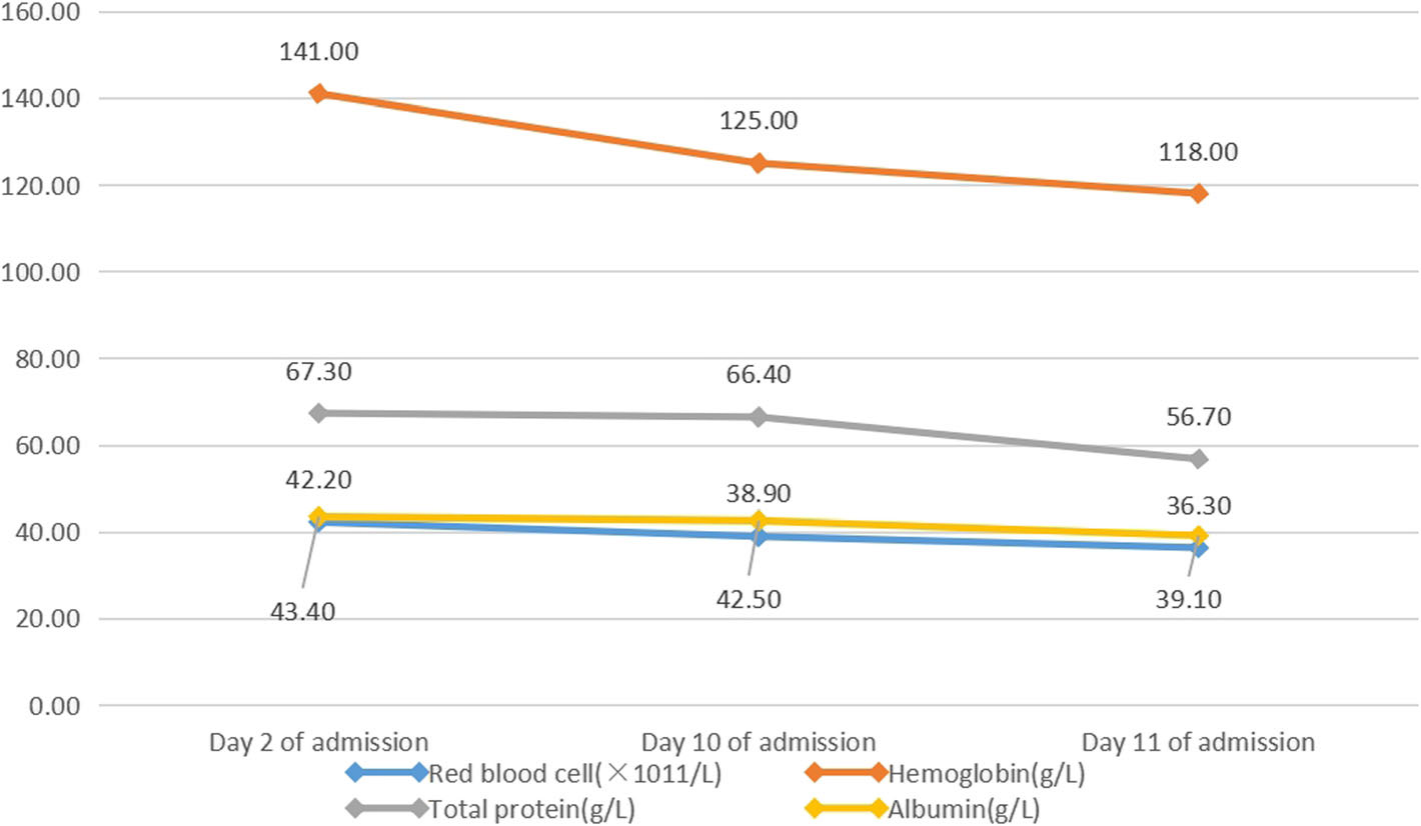
Preoperative nutrition index trend

Tumors consumption, surgical stimulation and increased postoperative consumption all could cause nutritional problems of patients [17–19]. The patient was vulnerable to malnutrition. At admission day, the NRS 2002 score of the patient was 1 point. On the 2nd to 10th day of admission, nutritional indicators such as red blood cells hemoglobin, total protein, albumin, etc. were in the normal range, but there was a downward trend (Fig. 4). Parenteral nutrition support was given according to the doctor’s advice. The patient was able to tolerant of surgery, although there existed a slight nutritional risk.

**Fig. 5:**
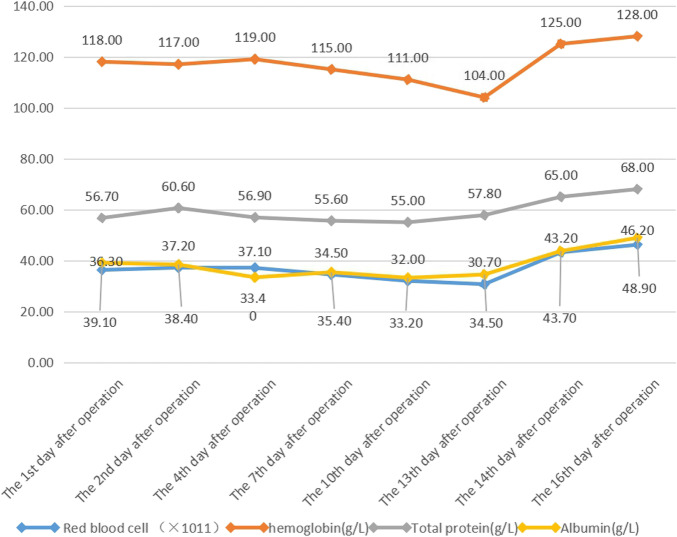
Postoperative nutritional index trend

After the operation, NRS2002 score of the patient was 3 point, which related to the restriction of fluid intake, negative balance, and limited intravenous nutrition supplement after left lung resection. The patient stayed in bed for a long time, resulting in poor body activity and gastrointestinal peristalsis, and inadequate diet. The medical care team and nutritionists had jointly formulated the patient’s postoperative diet and nutrition plan, evaluated the gastrointestinal function and adjusted the diet plan dynamically. Relating drugs were given to regulate the intestinal flora and to facilitate the recovery of gastrointestinal function. Nutritional assistance was also supported which included parenteral and oral nutrition, such as human albumin and semi-liquid digestible food. The nurse auscultated bowel sounds per shift and observed gastrointestinal symptoms. Until discharge, the nutritional status of the patient was stable; the results of the postoperative blood test are shown in the figure below (Fig. 5).

#### Psychosocial support

The diagnosis and treatment of cancer are stressful events, patients might face fears, uncertainties, distress, and psychosocial needs [20]. Among patients experiencing severe distress, 10% ~ 50% of them reported that their treatment results and quality of life were affected [21]. The patient and his families had anxiety and depression, so their psychosocial support was the focus of nursing [22]. Due to the complexity and uncertainty of the operation, patients and their families are seriously anxious during the perioperative period. Psychological counseling was provided at different stages, including mindfulness decompression [23]. Psychological experts consulted the family members to know their current perplexity and proving them the psychological counseling and positive incentives [24]. Gradually the patient gained confidence in the treatment and actively cooperated.

## Conclusion

This study reported an innovative surgical method, shared nursing experience and challenges. This was the first time to share the nursing experience of patients undergoing a specialized surgical procedure. The individualized and comprehensive nursing measures were formulated, based on clinical experience and patients’ conditions, and the patient finally got a good treatment effect. During the treatment, careful observation and intensive care were very important for the recovery of the patient’s postoperative pulmonary function, which were able to prevent the postoperative complications effectively.

## Data Availability

Not applicable.

## Abbreviations

ECMO: Extracorporeal Membrane Oxygenation
CT: Computed Tomography
SCC: Squamous Cell Carcinoma
ACC: Adenoid Cystic Carcinoma
PDS: Polydioxanone
ICU: Intensive Care Unit
ECG: Electrocardiogram
ARDS: Acute Respiratory Distress Syndrome
VV-ECMO: Veno-venous Extracorporeal Membrane Oxygenation
ACT: Activated Clotting Time
PS/CPAP: Personality Support/Continuous Positive Airway Pressure
FiO_2_: Fraction of inspiration O_2_
PEEP: Positive End-expiratory Pressure
COVID-19: Corona Virus Disease 2019
